# Recent trends on the implementation of reticular materials in column‐centered separations

**DOI:** 10.1002/jssc.202100849

**Published:** 2022-02-05

**Authors:** Katerina Fikarova, Edward Moore, Alma Nicolau, Burkhard Horstkotte, Fernando Maya

**Affiliations:** ^1^ Australian Centre for Research on Separation Science (ACROSS) School of Natural Sciences (Chemistry) University of Tasmania Tasmania Australia; ^2^ Department of Analytical Chemistry Faculty of Pharmacy in Hradec Králové Charles University Hradec Králové Czech Republic

**Keywords:** capillary electrochromatography, covalent organic frameworks, high‐performance liquid chromatography, metal‐organic frameworks, monoliths

## Abstract

Advances in the development of column‐based analytical separations are strongly linked to the development of novel materials. Stationary phases for chromatographic separation are usually based on silica and polymer materials. Nevertheless, recent advances have been made using porous crystalline reticular materials, such as metal‐organic frameworks and covalent organic frameworks. However, the direct packing of these materials is often limited due to their small crystal size and nonspherical shape. In this review, recent strategies to incorporate porous crystalline materials as stationary phases for liquid‐phase separations are covered. Moreover, we discuss the potential future directions in their development and integration into suitable supports for analytical applications. Finally, we discuss the main challenges to be solved to take full advantage of these materials as stationary phases for analytical separations.

**Article Related Abbreviations**
APTES: 3‐aminopropyltriethoxysilane
COF: covalent organic frameworks
HKUST: Hong Kong University of Science and Technology
TAMOF: triazole acid metal‐organic framework
UiO: Universitetet i Oslo
ZIF: zeolitic imidazolate framework

## 1 INTRODUCTION

Reticular chemistry is based on the linking of organic and inorganic molecular building units through strong bonds yielding crystalline extended structures, which are often porous [1, 2]. As a result, the pore size of such materials can be controlled by carefully selecting the building blocks for their synthesis [3]. A vast number of materials based on reticular design have been reported in the last decades [4, 5]. The most studied family of reticular materials are metal‐organic frameworks (MOFs) [6, 7], based on linking metal ions (or clusters) with organic ligands, creating open crystalline structures with permanent porosity and high surface areas. Besides MOFs, covalent organic frameworks (COFs) are porous organic crystalline materials, which have been gaining interest in the past years [8, 9]. Many novel COFs have been reported from the exploration of a variety of chemistries for linking different organic building blocks into porous crystalline networks.

The first analytical applications based on reticular materials relied on the use of MOFs as materials for sample preparation, or as stationary phases for chromatographic separation [10]. These applications were commonly based on the direct packing of MOFs crystals in column format [11]. However, due to the nonspherical shape and small size of MOF crystals, the incorporation of MOFs into suitable supports for chromatographic separation has been a popular topic in recent years [12, 13]. MOF crystals have been grown in a single step [14], or by sequential layer‐by‐layer growth [15, 16], on typical supports for chromatographic separation, such as beads and monoliths. The direct addition of MOF crystals in monolith polymerization mixtures has also been reported [13, 17]. A limitation of this approach is the potential nonhomogeneous MOF distribution along the column (e.g., by sedimentation of MOF crystals before the monolith polymerization is completed), or MOF crystal embedding within the polymer monolith structure. This makes that a significant part of the MOF crystals is not accessible and will not contribute to the subsequent chromatographic application.

In recent years, the application of COF materials as stationary phases for chromatographic separation has been gaining interest. Different covalent reactions have been adapted for the synthesis of COFs, the first examples being the reversible formation of boroxine and boronate ester bonds, and the Schiff base condensation of imines, hydrazones, and squaraines [18, 19, 20, 21]. In the past 5 years, the first reviews covering the applications of COFs in analytical chemistry have been published [22, 23, 24, 25].

In our previous review, we focused on the different approaches developed for MOF immobilization on suitable supports for analytical separations [26]. The most often employed immobilization methods to this date are the direct embedding of the already synthesized MOF crystals in the chromatographic support, or the in situ MOF growth either single‐step or layer by layer. In the present review, we have focused on the recent developments in this research topic (2019–2020), and expanding the scope to the emerging use of COFs for analytical separations. Future directions in the implementation of MOF and COF materials for analytical separations are discussed by the end of the review.

## 2 METAL‐ORGANIC FRAMEWORKS

Recent developments in the application of MOFs for column‐centered separations have been directed toward the exploration of novel MOFs for chiral stationary phases, strategies for the immobilization of MOFs on particles and monoliths, the introduction of hierarchical porosity, or the immobilization of proteins.

### 2.1 Design of novel MOFs for chiral separations

An emerging trend in the application of MOFs for column‐based separations is the use of chiral molecules as MOFs ligands, as well as the introduction of chiral selectors in the MOF structure. Hong Kong University of Science and Technology‐1 (HKUST‐1), also known as MOF‐199, is based on the coordination of Cu(II) with trimesic acid. This MOF has been implemented for the capillary electrochromatographic enantioseparation of five basic drugs [27]. A silica capillary was silanized using 3‐aminopropyltriethoxysilane (APTES) and functionalized with glutaraldehyde, which is subsequently oxidized with KMnO_4_ yielding carboxylic acid groups. The functionalized capillary was sequentially treated with solutions of Cu(II) and trimesic acid and was then filled with a solution containing both precursors, which enabled the growth of a homogenous coating of HKUST‐1 with a submicrometric thickness. Addition of the chiral selector (carboxymethyl‐β‐cyclodextrin) to the background electrolyte enabled enantioselective separation of five basic drugs. While resolution of the enantiomer mixture was not possible in bare capillaries, the π‐interactions between the trimesic acid ligand in the HKUST‐1 coating and the aromatic analytes as well as the interaction between π electrons and the potentially uncoordinated Cu(II) sites enhanced analyte retention and separation efficiency, obtaining baseline resolution.

TAMOF‐1 (triazole acid metal‐organic framework) is an enantiopure MOF with permanent porosity (1200 m^2^ g^−1^), and with good stability in both water and organic solvents [28]. TAMOF‐1 is based on the coordination of Cu(II) with an organic linker synthesized from l‐histidine (l‐2‐deaza‐2‐(4H‐1,2,4‐triazol‐4‐yl)histidine). TAMOF‐1 with a random particle size between 0.2 and 10 μm was directly packed in a standard HPLC column and worked as a highly versatile chiral stationary phase for the enantiomeric resolution of chiral organic molecules. TAMOF‐1 demonstrated to be a competitive new material for chiral separations, and the incorporation of this material into suitable supports will be required to outperform commercial columns today on the market. Potential approaches to improve the chromatographic performance could be the layer by layer, or in situ crystal growth, of TAMOF‐1 on imidazole functionalized polymer beads and monoliths.

To improve flow permeability of as‐synthesized MOFs as column‐packed stationary phases for HPLC, MOF crystals were mixed with silica gel (Daisogel, SP‐120‐7P) facilitating their packing with a total load of 37.5% MOF in 100 mm × 4.0 mm id stainless steel columns [29]. The addition of a silica gel filler enabled performance evaluation of a novel homochiral MOF based on (R)−3,3′‐bis(6‐carboxy‐2‐naphthyl)−2,2′‐dihydroxy‐1,1′‐binaphthyl for enantiomer separation. Using silica particles as fillers to assist MOF packings is a simple approach for the preliminary evaluation of novel MOFs as stationary phases for chromatographic separation. Nevertheless, once an MOF with promising performance has been discovered, the immobilization of this MOF onto an optimum support for chromatographic separation will be required.

### 2.2 Immobilization on particles

**FIGURE 1:**
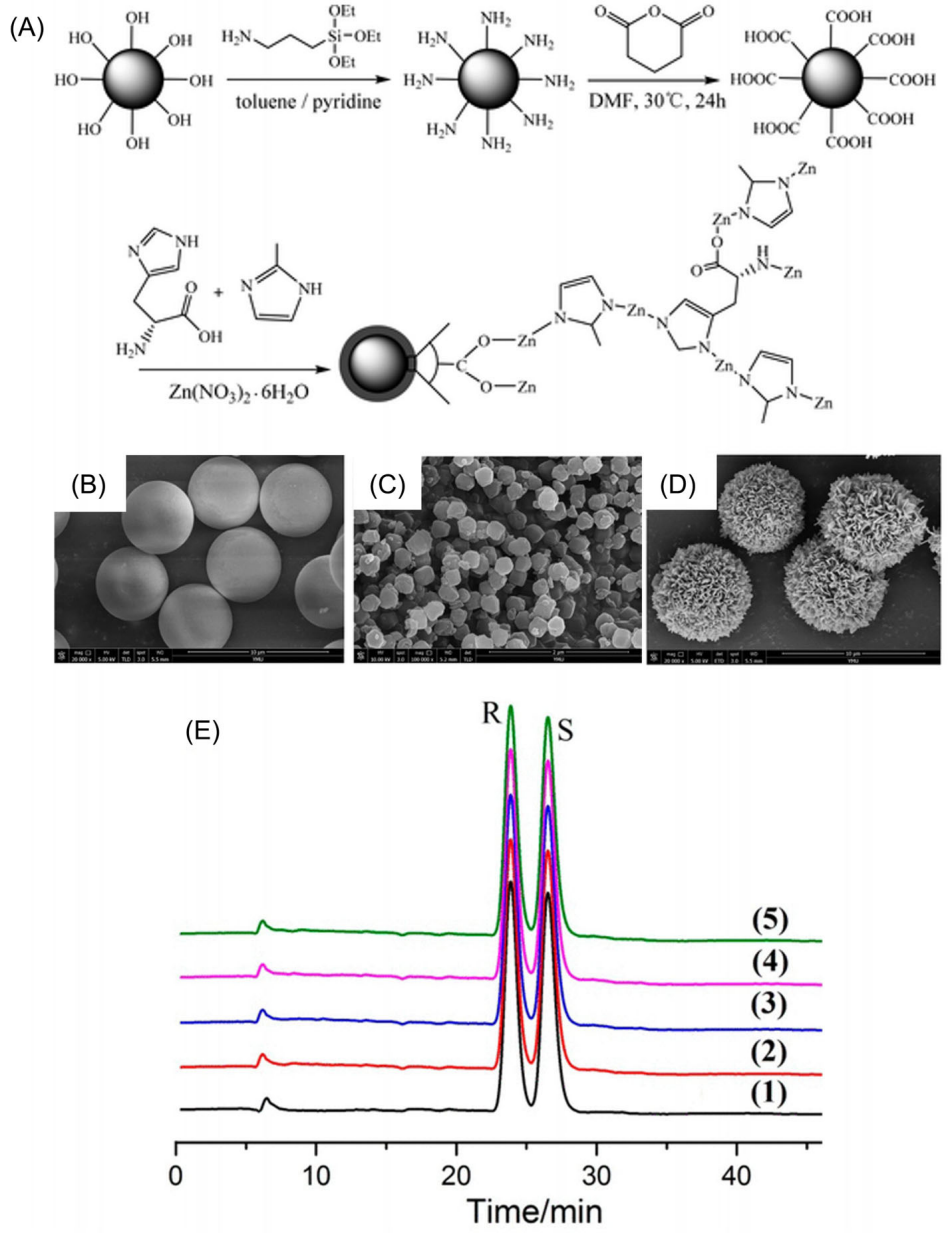
(A) Scheme for the preparation of d‐his‐ZIF‐8@SiO_2_ core–shell microspheres. SEM images of (B) SiO_2_–COOH, (C) d‐his‐ZIF‐8, and (D) d‐his‐ZIF‐8@SiO_2_. (E) Separation of trans‐stilbene oxide by repeated injections using a d‐his‐ZIF‐8@SiO_2_ stationary phase: (1) 50th injection, (2) 100th injection, (3) 150th injection, (4) 200th injection, and (5) 250th injection, respectively. Adapted with permission from Ref. [30]. Copyright (2020) American Chemical Society

Because of the above‐mentioned limitation, a histidine chiral MOF was grown in situ on silica beads with suitable shape and size for optimum HPLC packing performance [30]. This was achieved by silica functionalization with amino groups using 3‐aminopropyltriethoxysilane (Figure 1A). Thereafter, the amino groups were converted to carboxylic functional groups by the reaction with glutaric anhydride. Zeolitic imidazolate framework‐8 (ZIF‐8) was synthesized in situ in the presence of d‐histidine and sodium formate. The chiral properties of this stationary phase are attributed to the incorporation of the d‐histidine into the Zn(II)−2‐methylimidazole structure of ZIF‐8. The precursor SiO_2_–COOH microspheres featured a smooth surface and a particle size of approximately 5 μm (Figure 1B) making them ideal as HPLC packing. With an average particle size of approximately 200 nm, the d‐his‐ZIF‐8 crystals (Figure 1C) added about 0.5 μm to the final size of d‐his‐ZIF‐8@SiO_2_ particles (Figure 1D). The resulting packing showed to be highly robust with a very low RSD of 0.89% for the retention times of trans‐stilbene oxides over at least 250 injections (Figure 1E).

Silica particles have been used as a substrate to prepare core‐shell HPLC stationary phases based on the MOF UiO‐67 (Universitetet i Oslo‐67, Zr(IV)‐ 4,4′‐biphenyldicarboxylic acid) [31]. Here, commercial silica particles (5 μm diameter) with amino functional groups were selected as starting material. The starting silica was heated 120°C in dimethylformamide containing the dissolved metal precursor, followed by the addition of the organic linker. The reaction was continued for 24 h, and after washing the prepared stationary phase with methanol, a second MOF synthesis cycle was applied. The developed UiO‐67@SiO_2_ was used as a mixed‐mode hydrophilic interaction LC/reversed‐phase LC stationary phase and showed flexible selectivity for the separation of both hydrophobic (anilines, alkylbenzenes, and polycyclic aromatic hydrocarbons) and hydrophilic (thioureas) analytes. The main advantage in incorporating MOFs with organic linkers such as 4,4′‐biphenyldicarboxylic acid is the larger size of the linker molecule compared to the more common linker terephthalic acid. This will lead to a larger pore size in the synthesized MOF [32, 33], facilitating the analyte accessibility in the porous framework. However, a relevant factor here is the higher cost of linkers such as 4,4′‐biphenyldicarboxylic acid, with a cost around 200‐fold higher than terephthalic acid.

In situ growth of MOFs on silica particles has been carried out through the immobilization of sodium dodecyl benzenesulfonate, increasing the electrostatic interaction between the selected MOF precursors and the silica substrate [34]. Using this approach, MOF‐808 (Zr(IV)‐trimesic acid) was grown on mesoporous silica obtaining a stationary phase for hydrophilic interaction LC. Retention times proved to be stable for at least 120 h of continuous operation with RSDs in the range from 0.2 to 0.6% and a peak area repeatability in the range of 0.1 to 0.3%. A similar functionalization approach was used to grow bimetallic MOFs containing Zn(II) and Co(II), using 2‐methylimidazole as organic linker [35].

Another recent example of MOFs immobilization on silica is the in situ growth of the chiral MOF [Cu_2_((+)‐Cam)_2_Dabco] [36]. Silica was reacted first with Cu(OAc)_2_·2H_2_O to immobilize the MOF metal precursor, followed by reaction with d‐(+)‐camphoric acid and 1,4‐diazabicyclo[2.2.2]octane. To increase the MOF coating growth, the procedure was repeated twice. A second, or even a third, MOF growth cycle is usually described in these applications to fully cover the silica particles with MOF crystals. Normal phase chromatographic separations were achieved among different types of racemic compounds including carboxylic acids, ketones, and phenols.

### 2.3 Immobilization on monoliths

Presynthesized MOFs have been integrated into macroporous polymer monoliths allowing the straightforward use of MOFs as stationary phases in HPLC [13]. This approach was initially demonstrated with the copolymerization of a small amount of UiO‐66 crystals (Zr(IV)‐terephthalic acid) with methacrylic acid and ethylene dimethacrylate to yield a polymer monolithic stationary phase for reversed‐phase LC. An advantage of this approach is the straightforward implementation to other MOFs. This approach has been also implemented for the NH_2_‐MIL‐101 MOF (Al(III), Fe(III), or Cr(III) with 2‐aminoterephthalic acid as linker) in a glycidyl methacrylate‐ethylene dimethacrylate polymer monolith [37], leading to enhanced analyte retention due to additional hydrophobic and π–π interactions. However, other methods directed to growing the MOF crystals on the surface of the pores of the monolith may be more suitable, enhancing the contact between the MOF crystals and the analytes, and with a minimum change in the structure and mechanical stability of the monolith.

An alternative approach to combine MOFs and polymer monoliths relies in the synthesis of the polymer monolith followed then by a step‐by‐step growth of the MOF coating directly on the preformed monolith [16]. An example is the synthesis of a poly(glycidyl methacrylate)‐co‐(ethylene dimethacrylate) monolith, which is subsequently functionalized with imidazole groups using N‐(3‐aminopropyl)imidazole as reagent [38]. By sequential flushing of the imidazole‐functionalized monolith with the MOF precursor solutions (Zn(II) and 2‐methylimidazole), ZIF‐8 was grown directly on the monolith surface. The ZIF‐8 was used then as a substrate to covalently link pepsin as a chiral selector achieving a significant increase in surface against attaching the enzyme directly to the monolith. N‐(3‐Aminopropyl)imidazole was flushed through the final column leaving the ZIF‐8 crystals functionalized with amino groups. Amino groups were reacted with glutaraldehyde affording aldehyde functional groups enabling the immobilization of pepsin. The developed stationary phase was applied for the capillary electrochromatographic separation of six chiral drugs. In comparison with a pepsin functionalized poly(glycidyl methacrylate)‐co‐(ethylene dimethacrylate) in the absence of ZIF‐8, peak resolution was strongly enhanced for the tested racemic mixtures: hydroxychloroquine, 0.34 → 2.50; chloroquine, 0.45 → 1.97; hydroxyzine, 0.39 → 1.43; nefopam, 0.27 → 0.81; clenbuterol, 0 → 0.81; and amlodipine, 0.16 → 0.72.

**FIGURE 2:**
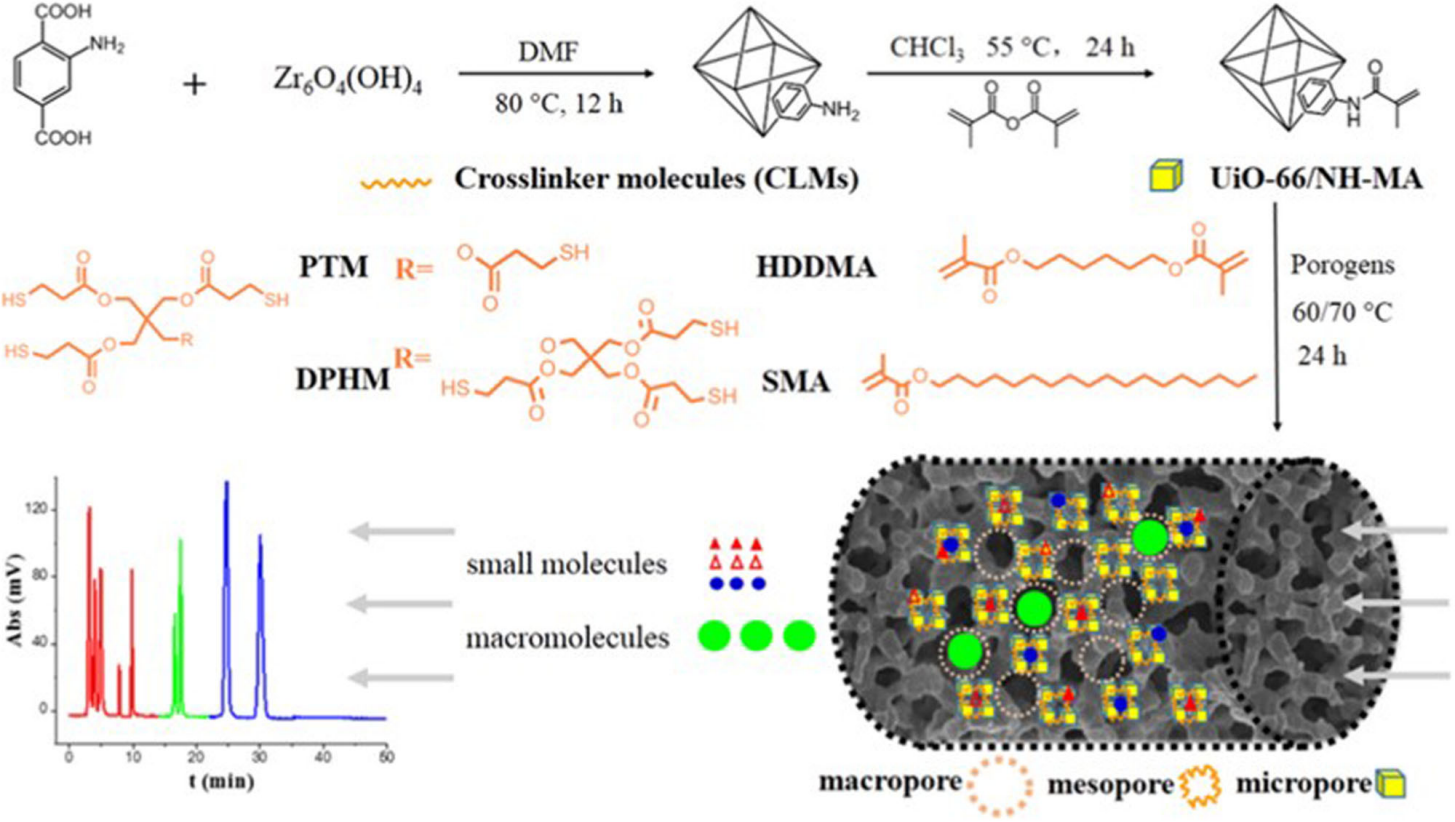
Methacrylic anhydride modification of NH_2_‐UiO‐66 and polymerization to obtain monolithic stationary phases with hierarchical porosity for the separation of small and large molecules. Reprinted with permission from Ref. [39]. Copyright (2020) American Chemical Society

The direct incorporation of MOFs in a polymer monolith has been improved by using the functional MOF linker 2‐aminoterephthalic acid [39] used for the synthesis of the MOF NH_2_‐UiO‐66. The amino group present in the organic linker does not participate in the coordination process with Zr(IV) but remains available for postsynthetic modification. In the reported work, the reaction of this amino group with methacrylic anhydride yielded a functionalization of the organic linker with vinyl groups obtaining polymerizable MOF crystals (**Figure** **2**) that were mixed with a cross‐linker, porogens, and initiator. Monoliths were synthesized in capillary columns and evaluated as stationary phase for nano‐HPLC. Monolithic columns containing the polymerizable MOF showed considerably lower HETP (28.1 μm) for ethylbenzene compared to the analogous polymer monolith without MOF (37.8 μm). This simple but effective approach can be extended to other MOFs based equally on the organic linker 2‐aminoterephthalic acid.

### 2.4 Hierarchical porosity

**FIGURE 3:**
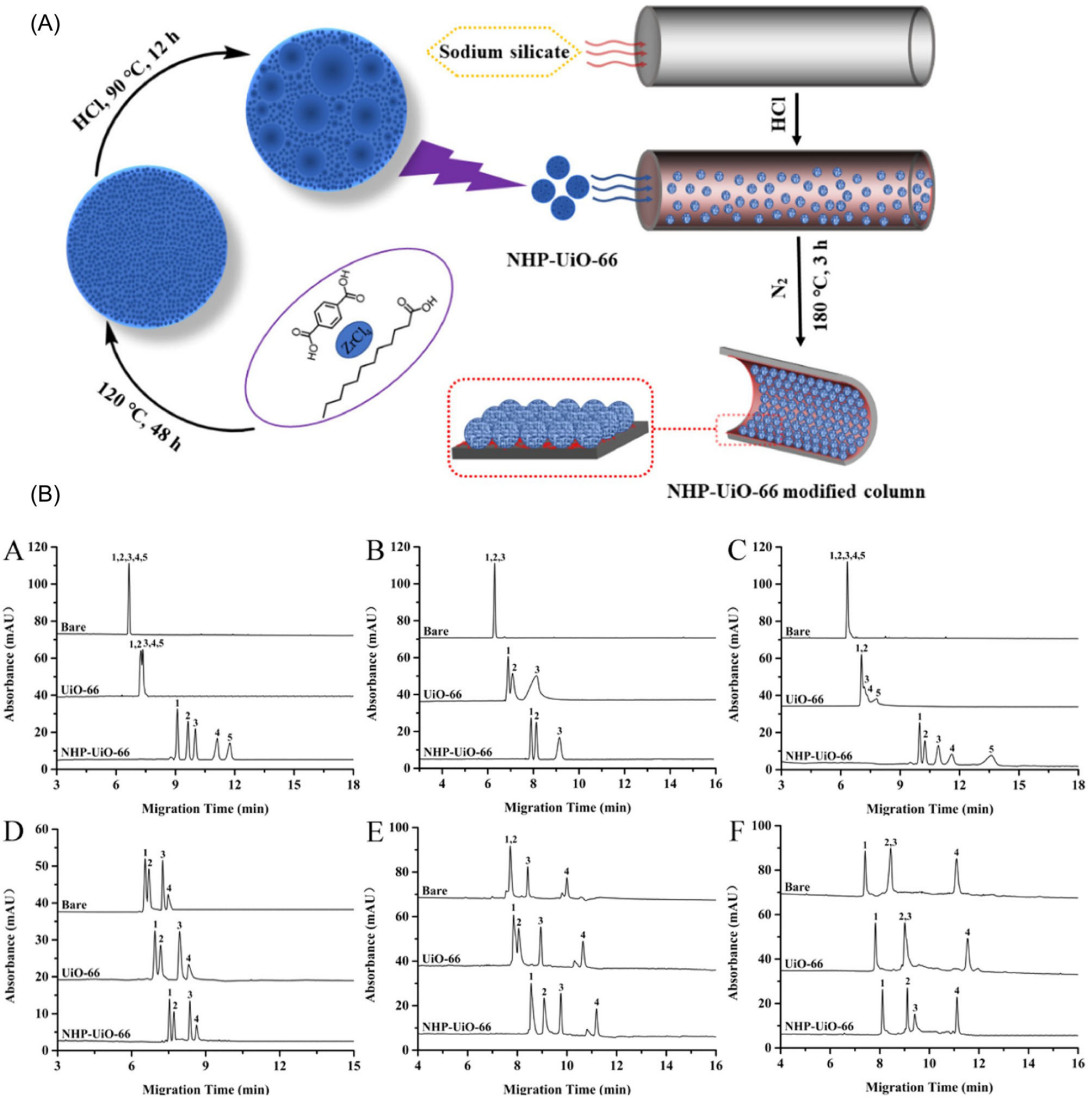
(a) Scheme for the preparation of NHP‐UiO‐66‐modified open‐tubular capillary column. (b) Electropherograms comparing bare, UiO‐66, and HP‐UiO‐66‐coated columns. (A) Substituted benzenes: (1) benzene, (2) methyl‐, (3) styrene, (4) ethylbenzene, (5) allylbenzene; (B) chlorobenzenes: (1) chlorobenzene, (2) *o*‐dichlorobenzene, (3) 1,2,4‐trichlorobenzene; (C) PAHs: (1) naphthalene, (2) acenaphthylene, (3) fluorene, (4) phenanthrene, (5) fluoranthene; (D) nucleosides: (1) cytosine, (2) cytidine, (3) uracil, (4) uridine; (E) polypeptides: (1) bacitracin, (2) colistin sulfate, (3) vancomycin, (4) thiostrepton; (F) basic proteins: (1) cytochrome *c*, (2) lysozyme, (3) myoglobin, (4) chymotrypsinogen. Adapted with permission from Ref. [43]. Copyright (2020) American Chemical Society

MOFs are predominantly microporous, and the very small size of MOF pores often limits the mass transport and diffusion of the analytes into the MOF sorbent/stationary phase, restricting the potential of MOFs in liquid chromatographic separations. To overcome this limitation, introduction of larger voids into MOF crystals has been attempted using polystyrene bead templates [40]. Based on this approach, higher analyte accessibility for sample preparation applications has been described [41] but such material has not been tested as a chromatography stationary phase yet. Alternatively, smaller voids can be introduced in the MOF crystal network by adding a modulator in the MOF synthesis [42]. This approach has been explored adding dodecanoic acid in the synthesis of the MOF UiO‐66 (Figure 3a) [43]. The resulting nanoscale hierarchically micro‐ and mesoporous MOF (NHP‐UiO‐66) was immobilized on the capillary inner surface and applied as stationary phase for capillary electrochromatography for substituted benzenes obtaining column efficiencies of up to 1.2 × 10^5^ plates/m with migration time RSDs lower than 5.8% for intraday, interday, and column‐to‐column comparisons. The separation performance of the so‐prepared NHP‐UiO‐66 column containing a hierarchical pore system was compared with an analogous column containing UiO‐66 crystals lacking hierarchical porosity as well as with a bare column. As shown in Figure, all tested analyte mixtures were baseline separated only when using the NHP‐UiO‐66 stationary phase. For nonpolar analytes, overlapping peaks were obtained when using the UiO‐66 stationary phase lacking the hierarchical pore system and separation failed completely for nonpolar analytes using the bare column.

### 2.5 Protein immobilization

The immobilization of ZIF‐8 crystals in a capillary has been used as substrate for the adsorption of the protein bovine serum albumin, benefiting from the multiple chiral binding sites of the protein for the subsequent application as a stationary phase for capillary electrochromatography [44]. The RSDs of run‐to‐run, day‐to‐day, column‐to‐column, and batch‐to‐batch reproducibility were all lower than 13.8%. The fast analysis of ephedrine isomers in Chinese herb ephedra was achieved with LODs of 1.5–2.0 ng mL^−1^ (S/N = 3) by applying an electrophoretic stacking technique of moving chemical reaction boundary. This example represents the possibility to use MOFs as supports for the immobilization of protein‐based stationary phases [45].

## 3 COVALENT ORGANIC FRAMEWORKS

Organic solids obtained by linking organic molecules via covalent bonds typically lead to amorphous materials. COFs are crystalline porous covalent solids composed entirely of light elements (B, C, N, O, Si) [8, 9, 46]. COFs are made by combining organic building units, covalently linked into an extended crystalline porous structure. Analytical applications using COFs as sorbents or stationary phases are recent, and these initial developments have been already compiled in several reviews [22, 25].

### 3.1 Immobilization on monoliths

**FIGURE 4:**
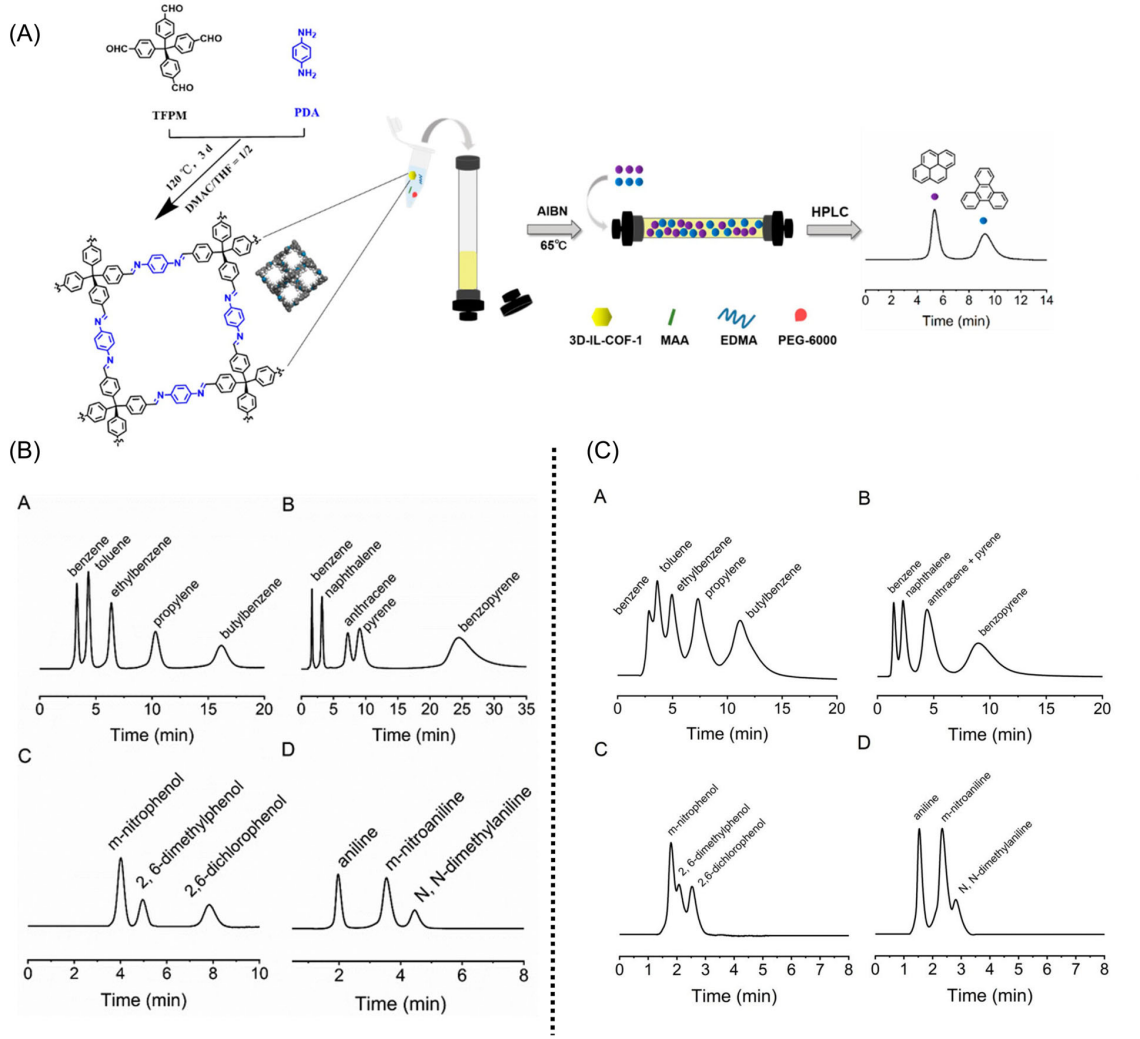
(A) Preparation of a covalent organic framework (COF)/polymer monolithic column for HPLC. HPLC chromatograms of neutral, acidic, and basic compounds on a (B) COF/polymer monolithic column and (C) blank polymer monolithic column, separated under identical chromatographic conditions. Adapted with permission from Ref. [47]. Copyright (2021) American Chemical Society

One of the implemented strategies for the incorporation of COFs in stationary phases for LC is the addition of presynthesized COFs into the polymerization mixture of polymer monoliths [47], an approach already used for the incorporation of MOFs on polymer monoliths [13]. The irregular shape and wide particle size distribution of COFs limits the direct application of these materials in the column‐packed format as stationary phases for HPLC. As an alternative, a COF/macroporous polymer monolith composite was prepared using tetrakis(4‐formylphenyl)methane and p‐phenylenediamine as building blocks (Figure 4A). The resulting material was obtained by polymerizing 15 mg of the COF material with methacrylic acid (35 μL), ethylene dimethacrylate (400 μL), and polyethylene glycol 6000 (450 mg), which were dissolved in dimethyl sulfoxide (1 mL) to obtain a stable and uniform dispersion. The prepared COF/polymer monolith had a significantly larger BET surface area (251 m^2^ g^−1^) than the pure polymer monolith (186 m^2^ g^−1^) owing to the incorporation of the larger BET surface area COF material (934 m^2^ g^−1^). The resulting COF/polymer monolith was synthesized in a column of 50.0 mm length × 4.6 mm id, and a blank column was also prepared using the same monolith precursors in the absence of COF particles. The performance of these columns as stationary phases for HPLC was compared, obtaining an improved separation performance for the COF/polymer monolith (Figure 4B), when compared with the COF‐free polymer monolith (Figure 4C).

Many of the COFs reported to date are based on the Schiff condensation of amines with aldehydes. A simple dialdehyde used for the construction of COFs is terephthalaldehyde, which has been combined with the amine heptakis(6‐amino‐6‐deoxy)‐β‐cyclodextrin to obtain a COF that enables the chromatographic separation of chiral drugs [48]. To immobilize a layer of the presynthesized COF particles in a capillary tube, the capillary was modified with γ‐methacryloxy propyl trimethoxysilane, a reagent commonly used to functionalize silica capillaries with vinyl functional groups. The COF crystals were dispersed (1 g/L) in a glycidyl methacrylate/methanol (10%, v/v) solution and the resulting mixture was heated (60°C, 6 h) to open the epoxy rings of the glycidyl methacrylate using unreacted amino groups of the COF. The initiator azobisisobutyronitrile was added to the mixture and the capillary was filled, polymerizing a thin layer of COF on the inner surface of the capillary. The prepared open tubular capillary column was applied to the separation of different enantiomeric drugs by capillary electrochromatography. While several enantiomeric drug mixtures were resolved using the cyclodextrin‐COF open tubular column, no separation was achieved by only functionalizing the capillary with glycidyl methacrylate, or by the capillary functionalized with heptakis(6‐amino‐6‐deoxy)‐β‐cyclodextrin. The inclusion of heptakis(6‐amino‐6‐deoxy)‐β‐cyclodextrin shaped a porous crystalline COF, contributing to the incorporation of greater amounts of cyclodextrin in the chromatographic support, as well as enhanced analyte accessibility toward the chiral selector. A similar example has been reported by using the same COF, but in this case, polymerizing a full monolith using glycidyl methacrylate and ethylene dimethacrylate [49]. The β‐cyclodextrin COF material–incorporated monolith achieved baseline separation for the selected amides, amino acids, nucleosides, aromatic acids, and positional isomers using capillary electrochromatography. In comparison with the original monolith, the β‐cyclodextrin COF material–incorporated monolith again showed significantly enhanced separation efficiency.

### 3.2 Packing

Silica particles have been used as fillers to enable column‐packings with COFs, similarly, as previously described with MOFs. A framework interpenetration strategy to synthesize a microporous COF for the efficient separation of C8 alkyl‐aromatic isomers has been recently reported. Two pairs of microporous 3D salen‐ and Zn(salen)‐based COFs were prepared by Schiff‐base condensation of ethanediamine with tetrahedral tetra(salicylaldehyde)‐silane or ‐methane derivatives in the presence or absence of metal ions [50]. To enable the application of this COF as a stationary phase for liquid chromatographic separation, the activated COF (550 mg) was crushed in methanol by applying soft pressure and mixed with ∼5 μm silica (200 mg) in 20 mL MeOH. The two salen‐COFs enabled baseline separation of xylene isomers and ethylbenzene with excellent column efficiencies and repeatability. Zn(salen)‐COFs were prepared by immobilizing Zn(II) on the ethanediamine building block of the salen‐COFs. While no improvement in the selected chromatographic separation was observed, this could be a potentially useful approach toward novel stationary phases for metal‐affinity separations.

### 3.3 Immobilization on particles

COFs have been incorporated into silica stationary phases through the functionalization of silica beads suitable for HPLC with amine groups using APTES, followed by reaction with terephthaldehyde to afford aldehyde groups [51]. The aldehyde functionalized silica was used as a substrate for the in situ growth of COF‐300 composed of terephthalaldehyde and tetra‐(4‐anilyl)‐methane. After packing the material in a stainless‐steel column, the separation of neutral and polar compounds was achieved in reverse phase chromatography mode due to hydrophobic interactions between the COF shell and the analytes. Proof of concept of neutral compounds separation in normal phase chromatography and hydrophilic phase chromatography modes were also reported.

### 3.4 Attachment to capillary surface

An azine‐linked COF based on 1,3,5‐tris(4‐formylphenyl)benzene and hydrazine was used to coat the internal surface of the capillaries and applied as a stationary phase for open‐tubular capillary electrochromatography [52]. The silica capillary was functionalized with epoxy groups via treatment with 3‐glycidoxypropyltrimethoxysilane. The capillary was filled with a solution of the presynthesized COF in methanol (2 mg/mL) and the capillary was sealed and heated at 70°C for 4 h. After washing the capillary using methanol and acetone, a thin layer of the COF material was immobilized through the remaining amine groups in the COF surface and the epoxy groups in the surface of the capillary after the functionalization step. The developed stationary phase was applied to the determination of bisphenol A and its analogues in beverage samples. A similar example based on a different COF synthesized using 1,3,5‐triformylphloroglucinol and 1,4‐phenylenediamine as building blocks was synthesized at room temperature and coated inside a capillary functionalized with APTES [53]. The presynthesized COF solution (2.5 mg/mL in acetonitrile) was loaded in the capillary, and in this case, the remaining aldehyde groups on the surface of the COF reacted with the amino groups of the functionalized capillary. Here, COF immobilization was carried out at room temperature.

The application of COFs as stationary phases in GC has been recently reported [54]. A hydrazone‐linked chiral COF has been covalently bonded onto the inner surface of fused silica capillaries functionalized with APTES. The COF was composed by 1,3,5‐benzenetricarboxaldehyde and the chiral building block (S)−2,5‐bis(2‐methylbutoxy)terephthalohydrazide. After filling the functionalized capillary with the precursor solution, COF solvothermal synthesis was carried out in the capillary, leaving behind a thin layer of COF covalently bound to the internal surface of the capillary. The COF coating exhibited good performance for GC separations of linear alkanes and alcohols, and aromatic positional isomers (nitrotoluene, benzenediol, naphthol, and xylene isomers).

## 4 FUTURE PERSPECTIVES

An interesting future direction toward improved column‐centered separations with advanced materials is 3D printing. Using fused deposition modeling 3D printing, filaments based on composite materials are re‐shaped into novel analytical devices for sample preparation. Combining thermoplastic filaments containing conductive carbons, or water‐soluble polymers, novel devices for membrane separation [55], electromembrane extraction [56], or SPE [57] have been reported. We have recently developed a composite filament based on acrylonitrile butadiene styrene and ZnO nanoparticles [58]. After 3D printing, the ZnO nanoparticles in the 3D‐printed part are reacted with 2‐methylimidazole, growing ZIF‐8 crystals on the available surface of the 3D‐printed device. The future development of composite filaments containing a water‐soluble polymer and reactive metal oxide nanoparticles is promising for the multimaterial fused deposition modeling of stationary phases with integrated macroporous polymers and immobilized microporous MOFs.

**FIGURE 5:**
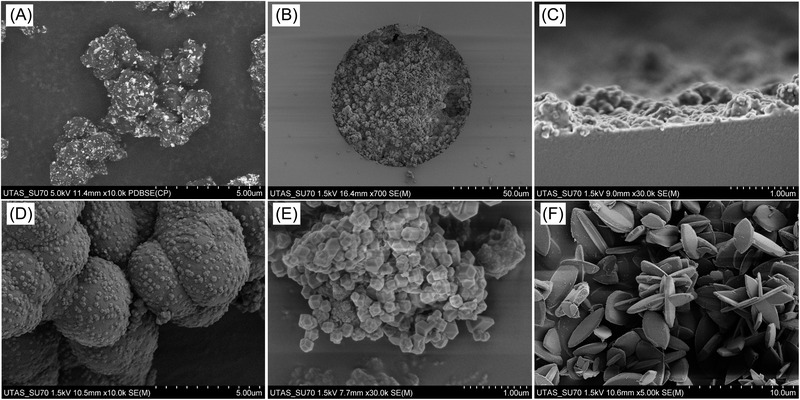
SEM images of poly(styrene‐co‐divinylbenzene) polymer monoliths containing 30 wt% of ZnO nanoparticles in bulk (A), capillary column (B), and open tubular column format (C). Bulk poly(styrene‐co‐divinylbenzene) polymer monolith (30 wt% of ZnO nanoparticles) after conversion to ZIF‐8 (D). Bulk poly(styrene‐co‐divinylbenzene) polymer monolith (30 wt% of ZnO nanoparticles) after conversion to ZIF‐8 in the presence of phenylalanine (E). Nonporous ZIF‐L crystals synthesized from ZnO nanoparticles (F)

Incorporation of ZnO precursors for MOF growth on column format supports has also been achieved on macroporous polymer monoliths [59]. In this initial application, ZnO nanoparticles were allowed to first react with methacrylic acid, and then cross‐linked with ethylene dimethacrylate using methanol and *n*‐dodecanol as porogens. Here, we worked with polymer monoliths containing up to 10 wt% load of ZnO nanoparticles. We recently tested the compatibility of commercial ZnO nanoparticles (https://www.sigmaaldrich.com/AU/en/product/aldrich/721085?60context=product) in other types of polymer monoliths, such as poly(styrene‐divinylbenzene) monoliths. We observed that commercial ZnO nanoparticle dispersions in ethanol can be added to poly(styrene‐co‐divinylbenzene) monoliths in a high load of up to 30 wt% (Figure 5A). This led to a large amount of the metallic precursor to grow Zn‐based MOFs, such as ZIF‐8. The poly(styrene‐co‐divinylbenzene) monolith material with 30 wt% ZnO nanoparticles can be easily adapted to capillary format, obtaining full monoliths (Figure 5B), or open tubular capillary columns (Figure 5C) by stopping the polymerization at an earlier stage (polymerization times were 6 h for the full monolith and 1 h for the open tubular column). After the precursor monolith synthesis, ZIF‐8 crystals can even be grown using aqueous solutions of 2‐methylimidazole after initial monolith swelling using methanol (Figure 5D). The in situ conversion of metal oxides is an efficient approach for MOF growth on separation supports, but the number of MOFs possible to grow following this approach is limited. The advantages and limitations of each method covered in this review for the application of porous crystalline materials for column‐based separations are listed in Table **1**.

**TABLE 1:** Different approaches for the implementation of porous crystalline materials (metal‐organic frameworks [MOFs]/covalent organic frameworks [COFs]) in column format

**Immobilization approach**	**Description**	**Advantages**	**Limitations**
Direct packing	Porous crystals directly packed in column	Simple. No further materials required	Packing limitations due to small size and/or nonspherical particle shape
Direct packing with filler	Porous crystals mixed with a filler and packed in column	Simple. Allows applications with smaller amounts of porous crystals	Contribution of the filler to analyte extraction/separation should be negligible. Potential nonhomogenous distribution of crystals throughout the column
Direct embedding in support	Porous crystals are added to the reagent mixture required to synthesize the separation support	Simple method to incorporate porous crystals on macroporous polymer monoliths	Large number of porous crystals buried in the polymer support. Small number of porous crystals active on the surface of the support for analyte extraction/separation
Single‐step growth	Porous crystals are synthesized in a single step on an already preformed support	Porous crystal growth is directed to the surface of the support being active for the subsequent extraction/separation application	The preformed separation support (beads, monolith) must have suitable functional groups to interact with one of the precursors of the selected MOF/COF. Several MOF/COF growth cycles might be required for a complete coating
Layer‐by‐layer growth	Porous crystalline coating growth by the sequential soaking of the separation support in the solutions of the required MOF/COF precursors	Porous crystal growth is directed to the surface of the support. Precise control over the properties of the coating	Limited number of MOFs/COFs grown with this approach. Time consuming, since many cycles are usually required to completely coat the support and develop a porous structure
In situ conversion of metal oxides	An MOF metallic precursor is embedded in the separation support followed by the in situ MOF growth by reaction with the selected organic linker	Fast procedure. Homogeneous coatings are grown in a single step with porous crystal growth directed to the surface of the support	Applicable to a limited number of MOFs. Metal oxide precursor must be homogeneously distributed in the separation support

Amino acids have been reported to act as crystallization agents in the synthesis of ZIF‐8 [60]. Depending on the selected amino acid, the size, shape, and yield of the obtained ZIF‐8 particles can be tuned. In a preliminary experiment, we tested the effect of adding the amino acid phenylalanine on the aqueous ZIF‐8 growth on poly(styrene‐co‐divinylbenzene) monoliths. As shown in Figure 5E, clear differences in the yield of the ZnO to ZIF‐8 conversion were observed when the amino acid was present. Amino acid modulation will be a useful tool for the fine tuning of the size and shape of MOF crystal growth on chromatographic supports. Additional possibilities in the development of advanced column‐based materials for analytical applications may be exploring the possibility of biomimetic mineralization in the MOF growth step [61].

Another field of interest is the exploration of the controlled introduction of defects in the MOF structures and their subsequent influence on the analytical performance of the material [62]. By exploiting defect engineering, supports for analytical sample preparation with enhanced analyte uptake and/or accessibility could be designed, including the development of more efficient stationary phases for chromatographic separation. While the role of defects in MOF crystals has been already explored in the design of novel catalysts [63], it has not yet been explored for analytical separations.

Characterization techniques such as inverse size exclusion chromatography could be beneficial to understand if the analytes are simply interacting with the surface of the MOF/COF crystals or penetrating the porous crystal network. In addition, comparison with analogous nonporous materials could be of interest. As a representative example, Zn(II) and 2‐methylimidazole can be used to synthesize a nonporous coordination polymer known as ZIF‐L (Figure 5F) [64]. Stationary phases containing either ZIF‐L or ZIF‐8 could be compared, enabling the study of potential additional effects related to the porous framework exclusive in ZIF‐8 for the selected application.

A limitation in the development of MOF applications is that most of these materials are prone to decompose in aqueous medium. This limitation is circumvented in the development of analytical applications using more stable MOFs based on high‐valent metals with carboxylate ligands (UiO MOFs) or combining low‐valent metals with imidazole‐based ligands (ZIFs). Nevertheless, even these types of more robust MOFs, such as the widely studied ZIF‐8, present a limited stability in buffer media. Despite ZIF‐8 being generally unstable in buffer media, with the initial crystalline framework being transformed into other different materials, or even dissolved in the presence of EDTA, ZIF‐8 showed stability in the presence of certain buffers [65, 66]. Methodologies to precisely monitor MOF stability in the development of analytical applications would be desirable. Precise measurements of MOF stability for pollutant extraction have been achieved by growing ZIF‐8 crystals in a highly stable ZnO‐CeO_2_‐Al_2_O_3_ mixed metal oxide [67]. The crystalline CeO_2_ phase is used as an internal standard enabling the stability monitoring of ZIF‐8 using powder X‐ray diffraction.

## 5 CONCLUSIONS

Porous crystalline materials, including MOFs and COFs, will continue creating new opportunities in the development of stationary phases for analytical separations. Special attention must be focused on the implementation of such crystalline materials into suitable supports to maximize chromatographic performance and moderate backpressure. To achieve this, it is critical to minimize channeling through nonideal packings by the immobilization of MOFs/COFs on monoliths and beads. This would overcome the potential size and shape limitations of MOF/COF crystals, minimize longitudinal diffusion, and enhance the analyte partition between the porous crystalline coating and the mobile phase. The development of MOF crystals with polymerizable ligands, or the introduction of defects in the MOF porous network are promising approaches toward the enhancement of the performance of these materials for chromatographic separations. Besides COF embedding in the stationary phase, COF shells in core–shell particles, or the attachment of pre‐synthesized COFs in functionalized capillaries, there are plenty of avenues for research in the development of analytical applications for COF‐based materials. It would be particularly interesting to see if pure COF monoliths (full columns or open tubular columns) can be synthesized in capillary format, and how these will perform as stationary phases for capillary LC.

## CONFLICT OF INTEREST

The authors have declared no conflict of interest.

## Data Availability

Data sharing is not applicable to this article as no new data were created or analyzed in this study.
